# A comprehensive analysis of the Cullin family reveals that CUL5 and CUL7 promote colorectal cancer progression and serve as prognostic markers

**DOI:** 10.1186/s41065-026-00677-8

**Published:** 2026-04-22

**Authors:** Noura Al-Dayan

**Affiliations:** Department of Medical Laboratory, College of Applied Medical Sciences, Prince Sattam bin Abdulaziz University, Al-Kharj, 11942 Saudi Arabia

**Keywords:** CRC, Cullin family genes, Prognosis, Diagnosis, Treatment

## Abstract

Colorectal cancer (CRC) remains a major cause of cancer-related mortality worldwide. Although Cullin family genes (CUL1, CUL2, CUL3, CUL4A, CUL4B, CUL5, CUL7, and CUL9) have been implicated in tumorigenesis, their roles in CRC are not fully defined. This study integrated in silico analysis with in vitro validation to characterize the expression and functional relevance of these genes in CRC. Transcriptomic profiling of TCGA datasets revealed significant up-regulation of CUL1, CUL2, CUL4A, CUL4B, CUL5, CUL7, and CUL9 in tumor tissues, with higher expression correlating with advanced stage and poorer survival, particularly for CUL5 and CUL7. Immunohistochemistry confirmed elevated protein levels, while genomic analysis showed that CUL9, CUL1, and CUL7 exhibited the highest mutation frequencies, though without significant survival impact. Single-cell and subtype analyses demonstrated distinct Cullin gene expression patterns across tumor-associated cell populations and immune or molecular subtypes. Functional enrichment indicated involvement in ubiquitin-mediated proteolysis and cancer-related pathways. Experimental knockdown of CUL5 and CUL7 in CRC cell lines significantly reduced proliferation and colony formation. ChIP-qPCR demonstrated increased H3K27ac enrichment at the CUL5 and CUL7 promoters in CRC cells, indicating epigenetic activation. Cycloheximide (CHX) chase assays further showed that silencing CUL5 or CUL7 accelerated the degradation of CREB1 and Cyclin D1, suggesting a role in substrate stabilization. Overall, our findings highlight the widespread up-regulation and functional significance of Cullin genes in CRC, with CUL5 and CUL7 emerging as key contributors to tumor progression and potential biomarkers or therapeutic targets.

**Supplementary Information:**

The online version contains supplementary material available at 10.1186/s41065-026-00677-8.

## Introduction

Colorectal cancer (CRC) is a prevalent and deadly malignancy, representing the third most common cancer worldwide and the second leading cause of cancer-related deaths [1, 2]. The burden of CRC is particularly high in developed countries, where lifestyle factors such as diet, obesity, and sedentary behavior contribute significantly to its incidence [3, 4]. However, low- and middle-income countries are experiencing a rapid increase in CRC cases, attributed to westernization of lifestyles and aging populations [5]. Advances in screening and early detection have improved survival rates in high-income regions, yet disparities in access to healthcare and screening programs persist, leading to late-stage diagnoses and poorer outcomes in less affluent areas [5]. The global landscape of CRC emphasizes the need for concerted efforts in prevention, early detection, and equitable access to treatment to combat this pervasive disease effectively.

One group of genes that has garnered attention in cancer research is the Cullin family [6, 7]. Cullins are a family of proteins that serve as scaffolds for the assembly of ubiquitin ligases, which are critical in the regulation of protein degradation via the ubiquitin-proteasome system [7, 8]. This system plays a vital role in maintaining cellular homeostasis by regulating the turnover of various proteins involved in cell cycle control, apoptosis, and signal transduction. Dysregulation of this pathway can lead to uncontrolled cell proliferation and tumorigenesis [9, 10].

Previous research has highlighted the involvement of Cullin family genes in various types of cancer. For instance, CUL1 has been implicated in the regulation of cell cycle progression and has been shown to promote tumor growth in several cancers, including breast and breast cancer [11]. Similarly, CUL3 is known to influence the degradation of key oncogenic proteins, and its aberrant expression has been associated with the development of renal cell carcinoma and melanoma [6, 12]. CUL4A and CUL4B have been studied for their roles in DNA damage response and chromatin remodeling, with overexpression observed in breast and hepatocellular carcinoma, respectively [13, 14]. Furthermore, CUL5, CUL7, and CUL9 have been reported to have diverse functions in tumor suppression and oncogenesis across different cancer types [15, 16].

Despite these advances, the specific roles of Cullin family genes in CRC remain underexplored. In particular, there is a paucity of comprehensive studies that integrate in silico and in vitro approaches to elucidate the diagnostic, prognostic, and therapeutic potential of these genes in CRC. Our study aims to fill this gap by conducting a detailed analysis of Cullin family gene expression in CRC, utilizing both computational [17, 18] and experimental methodologies [19].

## Methodology

### Cullin genes expression profiling across The Cancer Genome Atlas (TCGA) cohort

We downloaded the TCGA-CRC cohort data in Transcripts Per Million (TPM) format to analyze the expression of Cullin genes, encompassing 647 tumor cases and 51 normal cases. Data visualization was performed using the “ggplot2” package.

### Validation of Cullin genes expression across different clinical and demographic variables using additional TCGA cohort

UALCAN is an online resource for cancer data analysis, providing access to gene expression data from TCGA and other cancer datasets [18, 20]. Herein, UALCAN was used to validate the expression patterns of Cullin genes in COAD by comparing tumor and normal tissues and assessing variation across clinical and demographic subgroups. Analyses were performed using default UALCAN statistical settings, including differential expression assessed by Student t test with a significance threshold of p-value < 0.05.

### Proteomic expression profiling of the Cullin genes

The Human Protein Atlas (HPA) database is a comprehensive resource for exploring the protein expression and localization in human tissues and cells [21]. HPA was used in this work to assess protein level expression of Cullin genes by evaluating staining intensity, staining frequency, and subcellular localization across normal and COAD tumor samples. Analytical parameters included comparison of staining categories (not detected, low, medium, high), visual scoring based on antibody validation status.

### Prognostic values of the Cullin genes

The cSurvival database is an online tool designed for the survival analysis of cancer patients based on gene expression data [22]. It provides Kaplan Meier curves, hazard ratios, and statistical comparisons to assess gene outcome associations. Current study used cSurvival to determine the prognostic value of Cullin genes in COAD. Analyses were performed using median expression as the survival cut off, log rank testing with a significance threshold of p-value < 0.05, and Cox proportional hazards modeling to compute hazard ratios and confidence intervals.

### External validation of CUL5 and CUL7 expression and diagnostic performance

Expression validation of CUL5 and CUL7 was performed using the GEO dataset GSE44076 [23], which contains 98 colorectal tumor samples, 98 matched adjacent-normal mucosa samples, and 50 healthy colon mucosa samples. Raw expression matrices and sample annotation files were downloaded through the GEOquery package in R. Probe-level intensities were log2-transformed, background-corrected, and normalized using the limma pipeline. When multiple probes mapped to the same gene, expression values were collapsed using the average expression of corresponding probes. Samples were classified into “Tumor” and “Normal” groups based on GEO metadata, with normal samples including both adjacent normal and healthy mucosa tissues. Differential expression of CUL5 and CUL7 between tumor and normal groups was assessed using the Student’s t-test. Diagnostic performance was evaluated using ROC curve analysis implemented in the pROC package, and the area under the curve (AUC) was computed for each gene [24]. A combined diagnostic model was generated using a logistic regression classifier incorporating both CUL5 and CUL7 expression values. All analyses were conducted in R (version 4.3.0), and significance was set at *p* < 0.05.

### Mutational landscape of Cullin genes

The cBioPortal database is a comprehensive, open-access resource for exploring multidimensional cancer genomics data [25]. cBioPortal was employed to characterize the mutational landscape of Cullin genes in the TCGA COAD dataset. Analytical parameters included the use of GISTIC processed CNA data, mutation filtering based on nonsynonymous variants, alteration frequency thresholds above 1%, and visualization through OncoPrint and lollipop plots.

### Single-cell transcriptome data analysis

Tumor Immune Single-cell Hub 2 (TISCH2) is an scRNA-seq database that focuses on the tumor microenvironment (TME), offering comprehensive cell-type annotations at the single-cell level and enabling exploration of TME across various cancer types [26]. Single-cell expression data from the GEO GSE166555 dataset (12 CRC primary samples) were analyzed via TISCH2 to determine Cullin gene distribution across cancer-associated cell populations. Analytical parameters included quality control filtering based on gene count and mitochondrial gene percentage, normalization using default TISCH2 settings, differential expression assessment with adjusted p-value < 0.05, and visualization of gene distribution through UMAP embedding and cell cluster specific expression scores.

### Correlation analysis of Cullin genes with immune and molecular subtypes

TISIDB is an integrated repository for tumor and immune system interactions, offering a comprehensive resource for cancer immunology research [27]. In this work, TISIDB was used to assess the associations between Cullin gene expression and immune as well as molecular subtypes in COAD. Analytical parameters included correlation analysis using Spearman coefficients with a significance threshold of p-value < 0.05, subtype stratification based on predefined TISIDB classification schemes, and visualization through heatmaps and subtype specific expression plots.

### Correlation of Cullin genes with immune cells infiltration and drug sensitivity

The GSCA database is a robust platform designed for the comprehensive analysis of gene sets in the context of cancer [25]. In the present analysis, GSCA was used to evaluate the correlations of Cullin genes with immune cell infiltration and drug sensitivity in COAD. Analytical parameters included correlation analysis using Spearman coefficients with a significance threshold of p-value < 0.05, drug response evaluation based on normalized IC50 values from GDSC and CTRP datasets, and visualization through heatmaps and correlation matrices.

### Gene enrichment analysis

DAVID is a powerful bioinformatics tool designed to facilitate the functional annotation of large gene lists [28]. DAVID was employed to perform enrichment analysis of Cullin genes and identify associated biological processes and pathways. Analytical parameters included the use of default DAVID annotation categories, enrichment significance based on adjusted p-value < 0.05, and pathway selection using KEGG and GO terms with fold enrichment values above the platform threshold.

### Cell culture

Eight human colorectal cancer cell lines (HT-29, SW480, SW620, HCT116, Caco-2, LoVo, DLD-1, and RKO) were obtained from the American Type Culture Collection (ATCC, Manassas, VA, USA). Normal colonic epithelial cell lines CCD 841 CoN, FHC, CCD-112CoN, and CCD-33Co, as well as the normal colon fibroblast line CCD 18Co, were purchased from ATCC. The NCM460 cell line was obtained from INCELL Corporation (San Antonio, TX, USA). Primary human colon epithelial cells (HCoEpiC) were purchased from ScienCell Research Laboratories (Carlsbad, CA, USA). Cells were cultured in Dulbecco’s Modified Eagle Medium (DMEM) or RPMI-1640 supplemented with 10% fetal bovine serum (FBS) according to supplier recommendations. All cell lines were authenticated by short tandem repeat (STR) profiling and routinely tested for mycoplasma contamination prior to experimentation.

### RT-qPCR analysis

Total RNA from cells was collected when the cells reached 80% confluence to ensure optimal RNA yield and quality for analyzing the expression of Cullin genes. RNA extraction was meticulously performed using the SPARKeasy Cellular RNA Extraction Kit (AC0205, Shandong Sparkjade Biotechnology Co., Ltd.) [29], following the manufacturer’s protocol to ensure high purity and integrity of the RNA. The extracted RNA was then quantified using a spectrophotometer, and its quality was assessed by checking the A260/A280 ratio, aiming for a value close to 2.0 to confirm purity. Subsequently, cDNA synthesis was conducted with the Evo M-MLV RT Kit (AG11711, Accurate Biotechnology, Hunan, China). This process involved reverse transcription of 1 µg of total RNA in a 20 µL reaction mixture, incubated at 42 °C for 60 min, followed by inactivation of the reverse transcriptase at 85 °C for 5 min, as per the manufacturer’s instructions. The resulting cDNA was then diluted and stored at -20 °C until further use.

For quantitative PCR amplification, the Hieff^®^ qPCR SYBR Green Master Mix (11201ES08) was used. Each 20 µL PCR reaction mixture contained 10 µL of 2X SYBR Green Master Mix, 0.5 µL of each primer (10 µM), 1 µL of cDNA template, and 8 µL of nuclease-free water. The qPCR was performed on a real-time PCR system (CFX™, Bio-Rad). GAPDH was used as a reference gene for normalization to control for variations in RNA quantity and quality among samples. The relative expression levels of the Cullin genes were determined using the 2^−ΔΔCT^ method, where ΔCT represents the difference between the target gene and GAPDH CT values, and ΔΔCT represents the difference between the ΔCT of the sample and the control. The primers were sourced from a previous research [30] and their detail is given in Supplementary data Table 1.

### Knockdown of CUL5 and CUL7 genes

Small interfering RNAs (siRNAs) targeting CUL5 (5′-CAGCTGGTTATTGGAGTAAGA-3′) and CUL7 (5′-AUAAUCAGGACUACUCAACAUGUGC-3′) were obtained from Ruibo Company. Untransfected cells were used as the control group (Ctrl) to represent baseline physiological conditions without exposure to transfection reagents or synthetic RNA molecules.

Cells were seeded to achieve approximately 70% confluence at the time of transfection. siRNAs were delivered using Lipofectamine 2000 (Invitrogen, 11668) according to the manufacturer’s instructions. Briefly, siRNAs were diluted in Opti-MEM medium and incubated with Lipofectamine 2000 for 15–20 min at room temperature to allow complex formation before being added to the cells. After 6 h, the medium was replaced with fresh complete medium. Untransfected cells were selected as controls to avoid potential non-specific effects associated with lipid-based transfection or introduction of exogenous RNA, which may independently influence cellular stress responses, proliferation, or protein turnover pathways. Under our experimental conditions, transfection reagent treatment did not cause noticeable cytotoxicity or morphological alterations. Knockdown efficiency was confirmed 48 h post-transfection by RT-qPCR and Western blotting.

### Western blot analysis

Whole cell lysates were prepared using RIPA lysis buffer supplemented with a protease inhibitor cocktail and phosphatase inhibitors to preserve protein integrity. Lysates were incubated on ice for 20–30 min with intermittent vortexing, followed by centrifugation at 12,000 × g for 15 min at 4 °C to remove cellular debris. Protein concentrations were quantified using the BCA Protein Assay Kit, and equal amounts of protein (20–30 µg per sample) were mixed with 4× SDS loading buffer, boiled for 5 min, and separated by SDS–PAGE. Proteins were transferred onto PVDF membranes, which were then blocked for 1 h at room temperature with 5% BSA in TBST. Membranes were incubated overnight at 4 °C with the following primary antibodies: CUL5 Polyclonal Antibody (Thermo Fisher, PA5-114723), CUL7 Polyclonal Antibody (Thermo Fisher, A300-224 A), and GAPDH Recombinant Rabbit Monoclonal Antibody (Thermo Fisher, MA5-44674), used as an internal loading control. After TBST washes, membranes were incubated for 1 h at room temperature with HRP conjugated anti rabbit secondary antibodies. Protein bands were visualized using enhanced chemiluminescence (ECL) reagents and imaged on a digital chemiluminescence detection system. Densitometric analysis was performed using ImageJ under standardized exposure settings.

### Cell proliferation assay

Cell proliferation was assessed using the Cell Counting Kit-8 (CCK-8, ApexBio Technology, Houston, United States). For the CCK-8 assay, transfected cells were seeded in 96-well plates at 2000 cells per well. CCK-8 reagent was added at 0, 24-, 48-, 72-, and 96-hours post-inoculation, and absorbance at 450 nm was measured with a spectrophotometer.

### Colony formation assay

For the colony formation assay, 1000 cells were seeded in 6-well plates and incubated for 2 weeks. The cells were then fixed with ice-cold methanol, stained with crystal violet, and imaged. The colonies were quantified using the AlphaImager HP system (Alpha Innotech).

### Chromatin Immunoprecipitation (ChIP)-qPCR

Chromatin immunoprecipitation was performed using the Pierce™ Magnetic ChIP Kit (Thermo Fisher Scientific, Cat. No. 26157) according to the manufacturer’s instructions. Briefly, CRC cells (SW480 and HCT116) and normal colon epithelial cells (CCD 841 CoN) were cross-linked with 1% formaldehyde (Thermo Fisher) for 10 min and quenched with 125 mM glycine (Thermo Fisher). Nuclei were isolated and chromatin was enzymatically sheared using the kit’s Micrococcal Nuclease to obtain 200–500 bp fragments. Equal amounts of chromatin were incubated overnight at 4 °C with anti-H3K27ac antibody (Thermo Fisher Scientific, PA5-88099) or Normal Rabbit IgG (Thermo Fisher, Cat. No. 31235) as a negative control, followed by capture with magnetic protein A/G beads provided in the kit. Immune complexes were washed, eluted, and cross-links were reversed at 65 °C for 1–2 h using the kit’s elution buffer. DNA was purified using the Purification Columns included in the kit. Enrichment of the CUL5 and CUL7 promoter regions was quantified by qPCR using PowerUp™ SYBR™ Green Master Mix (Thermo Fisher). Percent input was calculated and normalized to IgG controls. All ChIP-qPCR experiments were performed with three biological replicates, each run-in technical triplicate.

### Cycloheximide (CHX) protein stability and half-life determination

Protein stability of CREB1 and Cyclin D1 was assessed using a CHX chase assay under the assumption of first-order degradation kinetics. SW480 and HCT116 CRC cells were seeded in 6-well plates and transfected with control siRNA (Ctrl), si-CUL5, or si-CUL7 using Lipofectamine 2000 (Thermo Fisher Scientific) according to the manufacturer’s instructions. Forty-eight hours post-transfection, cells were treated with 100 µg/mL CHX (Sigma-Aldrich, Cat. No. C4859) to inhibit de novo protein synthesis. Cells were harvested at 0, 2, 4, 6, and 8 h following CHX treatment. Whole-cell lysates were prepared using RIPA buffer supplemented with protease inhibitors. Protein levels of CREB1 and Cyclin D1 were quantified using ELISA kits (CREB1 Human ELISA Kit, Cat. No. KHO0231; Cyclin D1 Human ELISA Kit, Cat. No. A102931) according to the manufacturers’ protocols. Absorbance values were normalized to the 0-hour time point (set to 1.0) to determine relative protein abundance. To confirm exponential decay kinetics, normalized protein levels were log-transformed and plotted against time. Linear regression analysis of ln(relative protein abundance) versus time was performed to estimate the degradation rate constant (k). Protein half-life (t½) was calculated using the equation:

$$\mathrm{t}\frac{1}{2}=\mathrm{ln}\left(2\right)/\mathrm{k}$$

Goodness-of-fit was assessed by R² values of the log-linear regression model. Half-life values were calculated from at least three independent biological replicates and expressed as mean ± SD.

### Statistical analysis

All statistical analyses were performed using R software (version 4.2.3), SPSS (version 21.0), and GraphPad Prism (version 9). For comparisons between two groups, an independent-samples t test was used when data met assumptions of normality and homogeneity of variance. Normality was assessed using the Shapiro–Wilk test, and equality of variance was evaluated using Levene’s test. When these assumptions were violated, the Mann–Whitney U test was applied as a nonparametric alternative. For comparisons involving more than two groups, one-way ANOVA followed by Tukey’s post-hoc test was used for normally distributed data; otherwise, the Kruskal–Wallis test with Dunn’s post-hoc correction was performed. Correlations between gene expression and clinical or molecular features were analyzed using Pearson or Spearman correlation coefficients, depending on data distribution. Survival analyses were conducted using Kaplan–Meier curves, and statistical significance was determined using the log-rank test. Hazard ratios (HRs) and 95% confidence intervals (CIs) were calculated using Cox proportional hazards regression models. For in vitro experiments (RT-qPCR, CCK-8 assays, colony formation assays, wound healing, Western blot quantification, ChIP-qPCR, and CHX chase assay), each experiment included at least three independent biological replicates, and each biological replicate contained three technical replicates, unless otherwise specified. Data are presented as mean ± standard deviation (SD) unless indicated. Error bars displayed in all figures represent SD. P*-value < 0.05, P**-value < 0.01, and P***-value < 0.001 were considered indicative of statistical significance.

## Results

### Expression analysis of Cullin genes across The Cancer Genome Atlas (TCGA) cohort

**Fig. 1:**
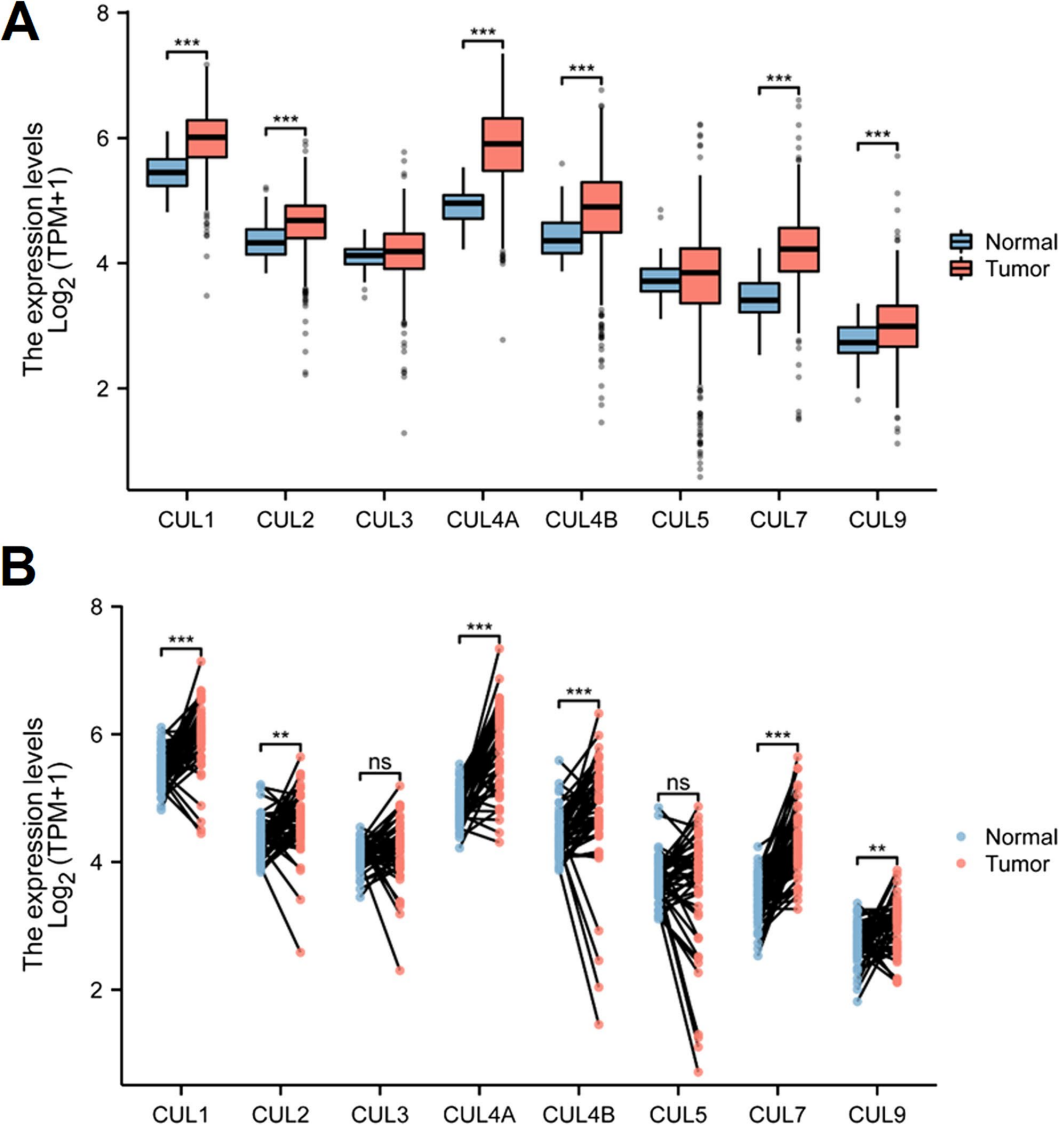
Differential mRNA expression of Cullin family genes in colorectal cancer (CRC) based on TCGA data. **A** Box plots showing mRNA expression levels of Cullin family genes (CUL1, CUL2, CUL3, CUL4A, CUL4B, CUL5, CUL7, and CUL9) in CRC tumor tissues (*n* = 647) versus normal tissues (*n* = 51) from the TCGA cohort. **B** Comparison of Cullin gene expression between paracancerous and tumor tissues from the same TCGA dataset. Group comparisons were performed using an independent samples t test. Data are presented as mean ± SD. P**-value < 0.01 and P***-value < 0.001

We examined the mRNA expression of Cullin family genes in CRC and normal tissues using data from the TCGA database. Our findings revealed that the expression levels of most genes, including CUL1, CUL2, CUL4A, CUL4B, CUL5, CUL7, and CUL9, were significantly elevated in CRC tissues (Fig. 1A). Further analysis of the expression patterns in paracancerous and tumor tissues from the TCGA database confirmed that CUL1, CUL2, CUL4A, CUL4B, CUL5, CUL7, and CUL9 were more highly expressed in tumor tissues relative to normal control tissues (Fig. 1B).

### Expression level of Cullin family genes across various clinical and demographic variables

**Fig. 2:**
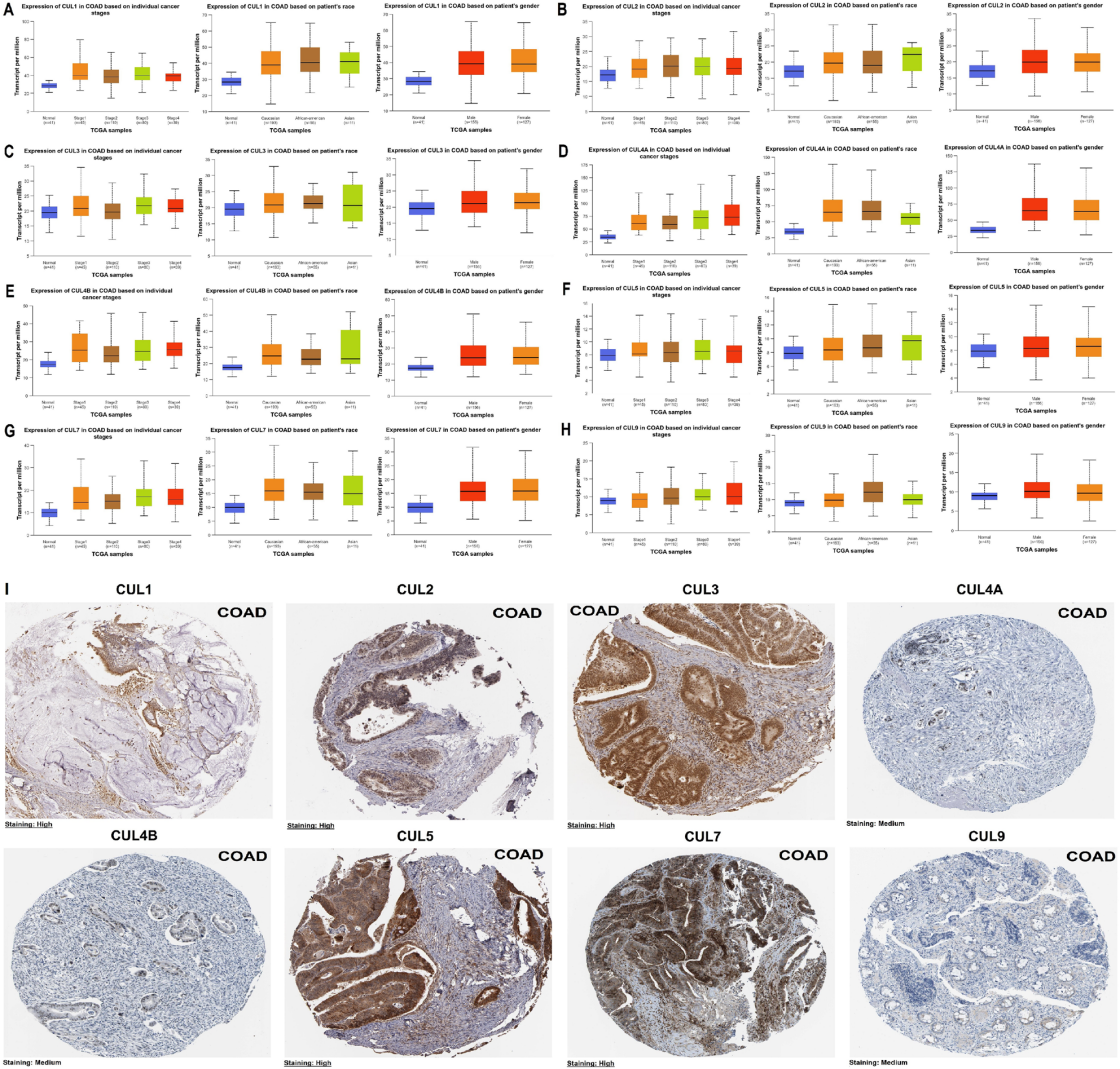
Expression levels of Cullin family genes across clinical and demographic variables in COAD and validation by immunohistochemistry. **A**–**H** Box plots showing expression levels of Cullin family genes in COAD versus normal tissues, stratified by tumor stage, race, and gender using UALCAN. Comparisons across multiple stages and racial groups were evaluated using one way ANOVA followed by Tukey post hoc tests; comparisons between male and female patients were analyzed using an independent samples t test. (I) Representative immunohistochemical staining images from the Human Protein Atlas (HPA) database. Data in panels A–H are presented as mean ± SD. P-value < 0.05

Figure 2 illustrated the expression levels of Cullin family genes in COAD tissues compared to normal tissues, stratified by cancer stages, patient race, and gender, based on data retrieved from the UALCAN database. The analysis revealed a general trend of increased expression of these genes in COAD tissues across all cancer stages (Fig. 2A–H). Additionally, racial differences in gene expression were observed, with Caucasian, Asian, and African-American patients exhibiting higher expression levels relative to normal controls. In contrast, there were no significant differences in gene expression between male and female patients, suggesting that gender did not influence Cullin gene expression in COAD. These findings indicated that Cullin family genes were upregulated in COAD and could potentially serve as biomarkers for the disease. Moreover, the results suggested that racial factors may influence expression variability, whereas gender did not appear to have a substantial effect. In addition, Fig. 2I presented immunohistochemical staining images from the HPA database, demonstrating the protein expression of the same Cullin family members in COAD tissues. The brown coloration indicated staining intensity, reflecting protein abundance. High expression levels of CUL1, CUL2, CUL3, CUL5, and CUL7 were evident based on strong staining intensity. In contrast, CUL4A, CUL4B, and CUL9 showed moderate staining, corresponding to intermediate levels of protein expression in CRC tissues.

### Mutational analysis of Cullin genes

**Fig. 3:**
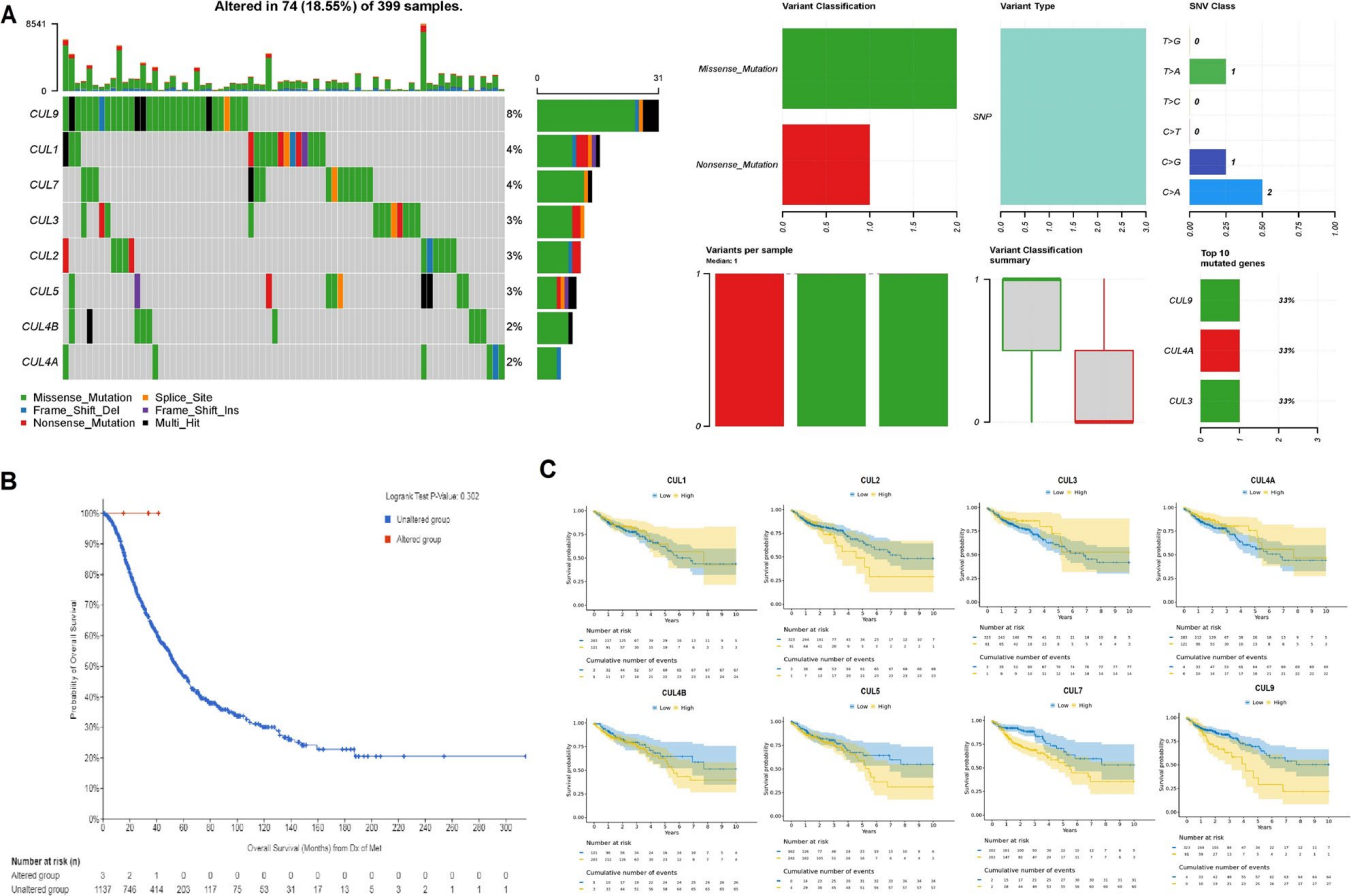
Mutational landscape of Cullin family genes in colorectal cancer (CRC) and their impact on overall survival. **A** Summary of genetic alterations in Cullin genes across 399 CRC samples in cBioPortal, including mutation frequencies and alteration types (missense, nonsense, frameshift, and other variants). **B** Kaplan–Meier survival curve comparing overall survival in CRC patients with alterations in Cullin genes versus those without alterations. **C** Kaplan–Meier survival curves showing overall survival in CRC patients stratified by high versus low expression (median cut off) of individual Cullin genes. Survival differences in panels **B** and **C** were evaluated using the log rank test, and hazard ratios with 95% confidence intervals were obtained from Cox proportional hazards models. P-value < 0.05

In CRC, the mutational landscape of Cullin genes was analyzed using data from the cBioPortal database. Figure 3A illustrated the distribution and types of genetic alterations across 399 CRC samples, revealing that 74 samples (18.55%) harbored alterations in Cullin genes. Among these, CUL9 emerged as the most frequently altered gene (8%), followed by CUL1 and CUL7, each exhibiting alterations in approximately 4% of the samples (Fig. 3A). The types of genetic alterations identified included missense mutations, frameshift deletions, and nonsense mutations, with the majority being single nucleotide polymorphisms (SNPs). The variant classification summary demonstrated that missense mutations were the predominant type of alteration, with a notable portion comprising nonsense mutations (Fig. 3A). Figure 3B presented a Kaplan–Meier survival curve, comparing the overall survival of CRC patients with alterations in Cullin genes to those without such alterations. The survival analysis revealed no statistically significant difference in overall survival between the two groups, as indicated by a Logrank test p-value of 0.302 (Fig. 3B). These findings suggested that, although Cullin gene mutations were present in a subset of CRC patients, they did not significantly influence overall survival outcomes within this cohort.

### Prognostic values of the Cullin genes

In this study, the cSurvival database was utilized to evaluate the prognostic significance of Cullin genes in CRC. Kaplan–Meier survival curves demonstrated that high expression levels of CUL1, CUL2, CUL3, CUL4A, CUL4B, CUL5, CUL7, and CUL9 were associated with poorer survival outcomes in CRC patients (Fig. 3C). However, upon statistical evaluation, significant prognostic value was identified only for CUL5 and CUL7 (Fig. 3C). Patients with elevated expression of these two genes exhibited significantly reduced overall survival compared to those with lower expression levels (Fig. 3C). These findings suggested that CUL5 and CUL7 may serve as reliable prognostic biomarkers in CRC. Overall, the results indicated that while many Cullin genes showed trends toward adverse prognostic impact, CUL5 and CUL7 stood out as statistically significant indicators of poor prognosis.

### Validation of CUL5 and CUL7 expression using GEO dataset (GSE44076)

**Fig. 4:**
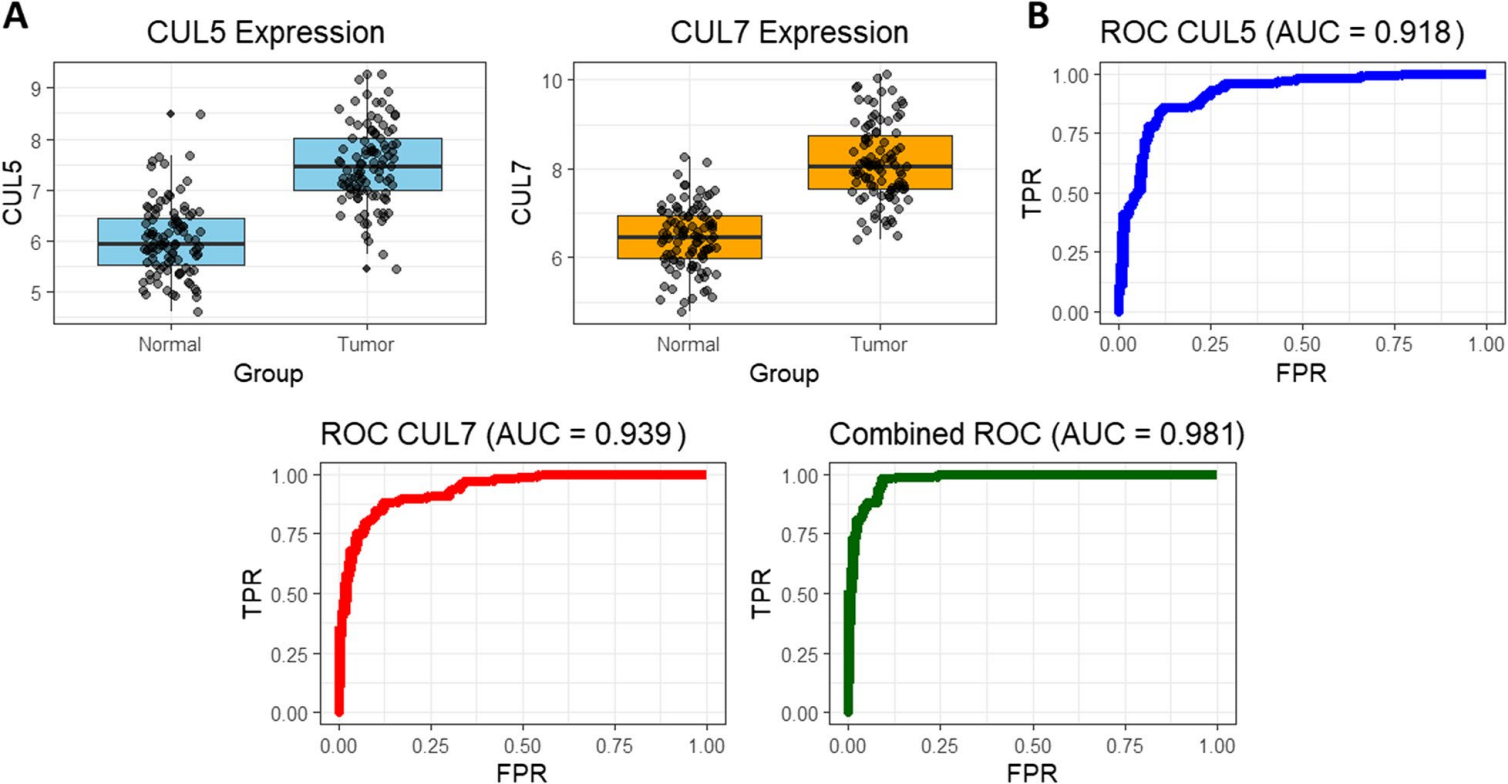
Validation of CUL5 and CUL7 expression and diagnostic performance using the GEO GSE44076 dataset. **A** Boxplot analysis of CUL5 and CUL7 expression levels in the GSE44076 cohort, comprising 98 colorectal tumor samples, 98 matched adjacent-normal mucosa samples, and 50 healthy colon mucosa samples. Both genes were significantly upregulated in tumor tissues compared with adjacent normal and healthy controls. Statistical significance was assessed using the Student’s t-test, with p-value < 0.05 considered significant. **B** ROC curve analysis evaluating the diagnostic performance of CUL5 and CUL7. CUL5 yielded an AUC = 0.842, CUL7 yielded an AUC = 0.879, and a combined logistic regression model incorporating both genes achieved an improved AUC = 0.912. ROC curves were generated using the pROC package in R, and 95% confidence intervals were calculated

To validate the differential expression of CUL5 and CUL7, we analyzed the independent GEO dataset GSE44076, which includes 98 colorectal tumor samples, 98 matched adjacent-normal mucosal samples, and 50 healthy colon mucosa samples. Consistent with our primary findings, both genes were significantly upregulated in CRC tissues. CUL5 expression was markedly elevated in tumors compared with adjacent normal and healthy controls (p-value < 0.05), and CUL7 showed a similar significant increase (p-value < 0.05) (Fig. 4A). ROC curve analysis demonstrated strong diagnostic performance, with CUL5 achieving an AUC of 0.842 and CUL7 an AUC of 0.879 for distinguishing tumor from non-tumor samples (Fig. 4B). A combined logistic regression model incorporating both markers further improved diagnostic accuracy, yielding a combined AUC of 0.912 (Fig. 4B).

### Correlations of Cullin genes with immune and molecular subtypes of CRC

**Fig. 5:**
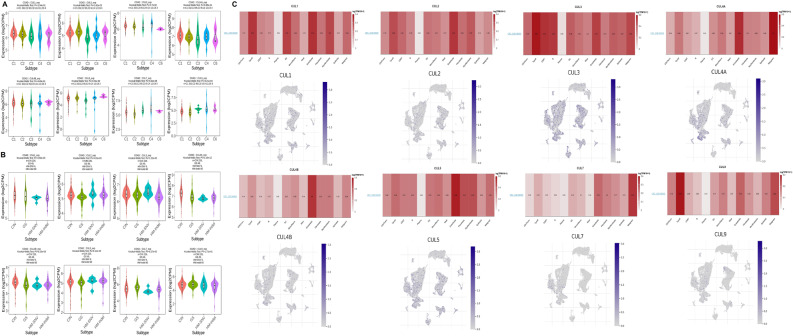
Correlations of Cullin gene expression with immune and molecular subtypes of colorectal cancer (CRC) and associations with tumor microenvironment (TME) cell populations. **A** Expression patterns of Cullin genes across consensus immune subtypes (C1–C6, excluding C5) in CRC, as obtained from TISIDB. **B** Expression profiles of Cullin genes across CRC molecular subtypes (CIN, GS, HM SNV, and HM Indel). Multi group comparisons in panels A and B were analyzed using one way ANOVA followed by Tukey post hoc tests. **C** Single cell RNA sequencing analysis of 12 CRC cases from GSE166555, showing the distribution of Cullin gene expression across 13 annotated cell populations, including B cells, conventional CD4 T cells, CD8 T cells, dendritic cells, endothelial cells, epithelial cells, fibroblasts, malignant cells, mast cells, monocytes/macrophages, myofibroblasts, plasma cells, and proliferating T cells. Data in panels **A** and **B** are presented as mean ± SD. P-value < 0.05

Correlations between Cullin gene expression and distinct immune and molecular subtypes of CRC were assessed using the TISIDB database. Figure 5A illustrated Cullin gene expression across five consensus immune subtypes (C1 to C6, excluding C5), revealing variable expression patterns among the gene family. Notably, CUL1, CUL4A, and CUL7 showed elevated expression in the C1 (wound healing) and C4 (lymphocyte depleted) immune subtypes, whereas CUL3, CUL4B, and CUL5 exhibited higher expression in the C6 (TGF-β dominant) subtype. Figure 5B presented the expression profiles of Cullin genes across four molecular subtypes of CRC (CIN, GS, HM-SNV, and HM-Indel). Distinct patterns were again observed, with CUL1 and CUL9 showing the highest expression in the CIN subtype, while CUL7 expression was predominantly elevated in the HM-SNV subtype.

### Correlations between tumor microenvironment (TME) and Cullin genes expression

Given that the dysregulation of Cullin genes expression contributes to an imbalance of immune components and consequently to the adverse progression of malignant tumors, we speculated that Cullin members might alter the ratio of cells in the TME. To investigate this, we analyzed single-cell transcriptome data from 12 CRC cases in the GSE166555 database. Using reported marker genes, we identified 13 cell subpopulations, including B cells, conventional CD4 T cells, CD8 T cells, dendritic cells, endothelial cells, epithelial cells, fibroblasts, malignant cells, mast cells, monocytes/macrophages, myofibroblasts, plasma cells, and proliferating T cells (Fig. 5C). The results indicated that most Cullin genes were associated with these cell types to varying degrees.

### Functional enrichment of Cullin genes

**Fig. 6:**
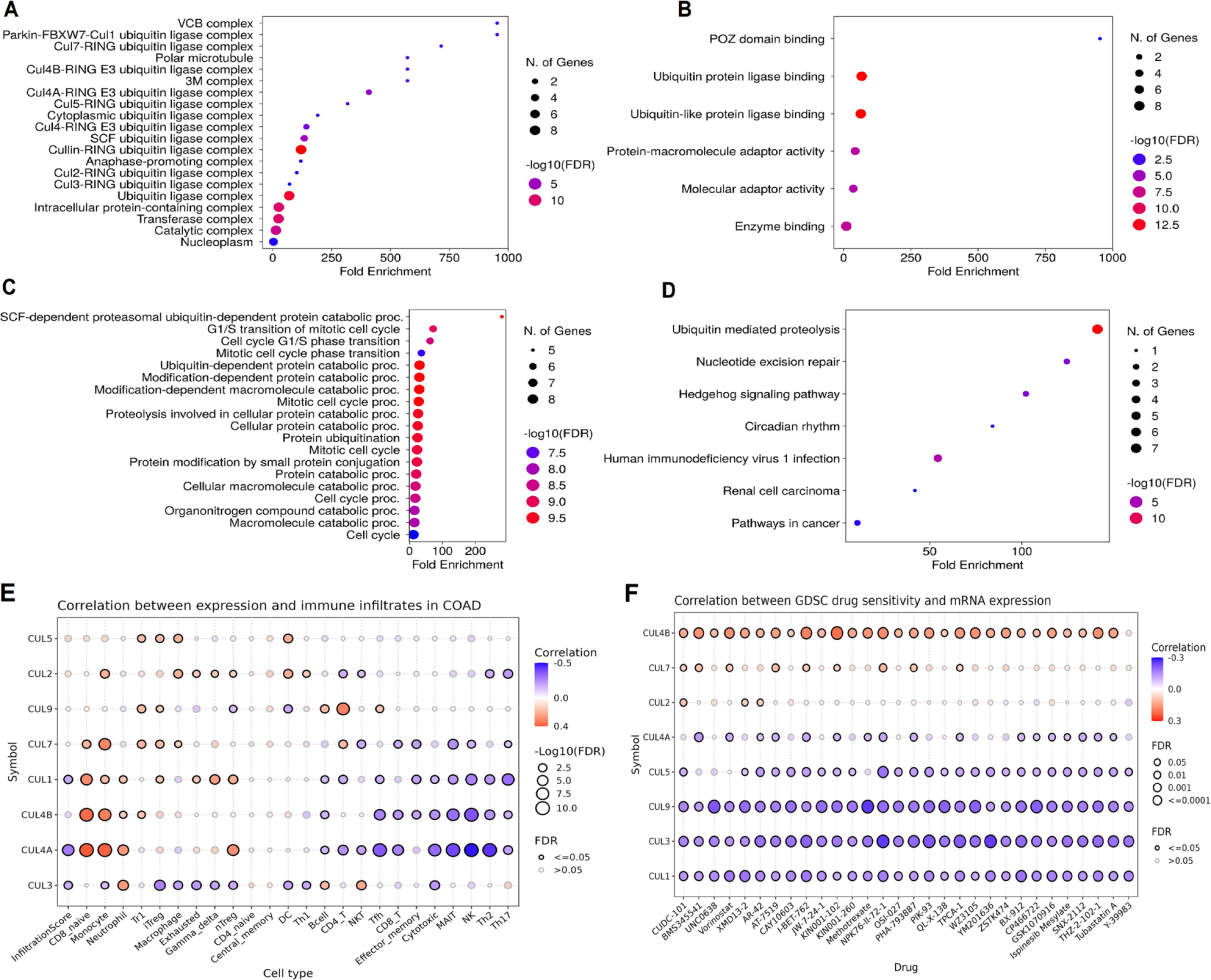
Functional enrichment analysis of Cullin family genes and their correlations with immune cell infiltration and drug sensitivity in CRC. **A**–**C** DAVID based Gene Ontology enrichment analysis of Cullin genes, showing significantly enriched cellular component (CC), molecular function (MF), and biological process (BP) terms. Bars represent fold enrichment, and term significance is indicated by adjusted P values. **D** KEGG pathway enrichment analysis highlighting pathways significantly associated with Cullin genes, including ubiquitin mediated proteolysis, nucleotide excision repair, and signaling pathways related to cancer. Enrichment analyses in panels **A**–**D** were with significance defined as adjusted *P* < 0.05 (Benjamini–Hochberg correction). (**E**) Heatmap displaying correlations between Cullin gene expression and immune cell infiltration in COAD based on GSCA. Colors indicate Spearman correlation coefficients (red, positive; blue, negative), and dot size represents statistical significance (− log10 FDR). **F** Heatmap showing correlations between Cullin gene expression and drug sensitivity (IC50 values) from the GDSC dataset. Colors indicate Spearman correlation coefficients (red, positive correlation suggesting resistance; blue, negative correlation suggesting sensitivity), and dot size reflects significance (− log10 FDR). Correlation analyses in panels E and F were performed using Spearman correlation. False discovery rate (FDR) adjusted p-value < 0.05 was considered statistically significant

Functional enrichment analysis of Cullin genes was conducted using DAVID tool. Figure 6 presents the results of gene enrichment analysis across various categories: cellular component (CC), molecular function (MF), biological process (BP), and KEGG (Kyoto Encyclopedia of Genes and Genomes) pathways. In Fig. 6A, the CC analysis shows significant enrichment of Cullin genes in” ubiquitin ligase complexes and related complexes, with the highest fold enrichment observed in the VCB complex, followed by various RING ubiquitin ligase complexes and the nucleoplasm.” In Fig. 6B, the MF analysis highlights significant enrichment in “ubiquitin protein ligase binding and related activities, such as ubiquitin-like protein ligase binding and molecular adaptor activities.” In Fig. 6C, the BP analysis reveals strong enrichment in processes related to the “ubiquitin-dependent protein catabolic process, including SCF-dependent proteasomal activity and various stages of the cell cycle (G1/S transition, mitotic cell cycle).” In Fig. 6D, the KEGG pathway analysis shows significant enrichment in “ubiquitin-mediated proteolysis, nucleotide excision repair, and several signaling pathways, including Hedgehog signaling, circadian rhythm, and pathways in cancer.”

### Correlations of Cullin genes with immune cells infiltration and drug sensitivity

Correlations between immune cell infiltration and drug sensitivity in COAD were investigated using data from the GSCA database. As shown in Fig. 6E, the heatmap illustrated the strength and significance of correlations between Cullin family gene expression and immune infiltrates in COAD. The color gradient represented the direction and magnitude of correlation (red indicating positive, blue indicating negative), while the dot size reflected statistical significance, represented as -log10(FDR). Among the notable findings, CUL4A and CUL4B exhibited significant negative correlations with multiple immune cell types, including CD8 + T cells, MAIT cells, and Th2 cells. These results suggested that CUL4A and CUL4B may negatively influence immune cell infiltration, potentially contributing to an immunosuppressive tumor microenvironment in COAD. In addition, Fig. 6F presented a heatmap showing the correlation between CUL gene expression and sensitivity to various drugs based on GDSC data. Drugs were displayed on the x-axis and Cullin genes on the y-axis. Correlation coefficients were color-coded, with red indicating positive correlation (potential drug resistance) and blue indicating negative correlation (potential sensitivity). Dot size again represented statistical significance. Notably, CUL4B expression was positively correlated with resistance to several drugs, including BM-553, Vincristine, and Vorinostat, indicating that higher levels of CUL4B may contribute to drug resistance. In contrast, CUL1 and CUL3 showed significant negative correlations with a range of drugs, suggesting that their elevated expression might be associated with increased drug sensitivity.

### Validation of Cullin genes expression in cell lines

**Fig. 7:**
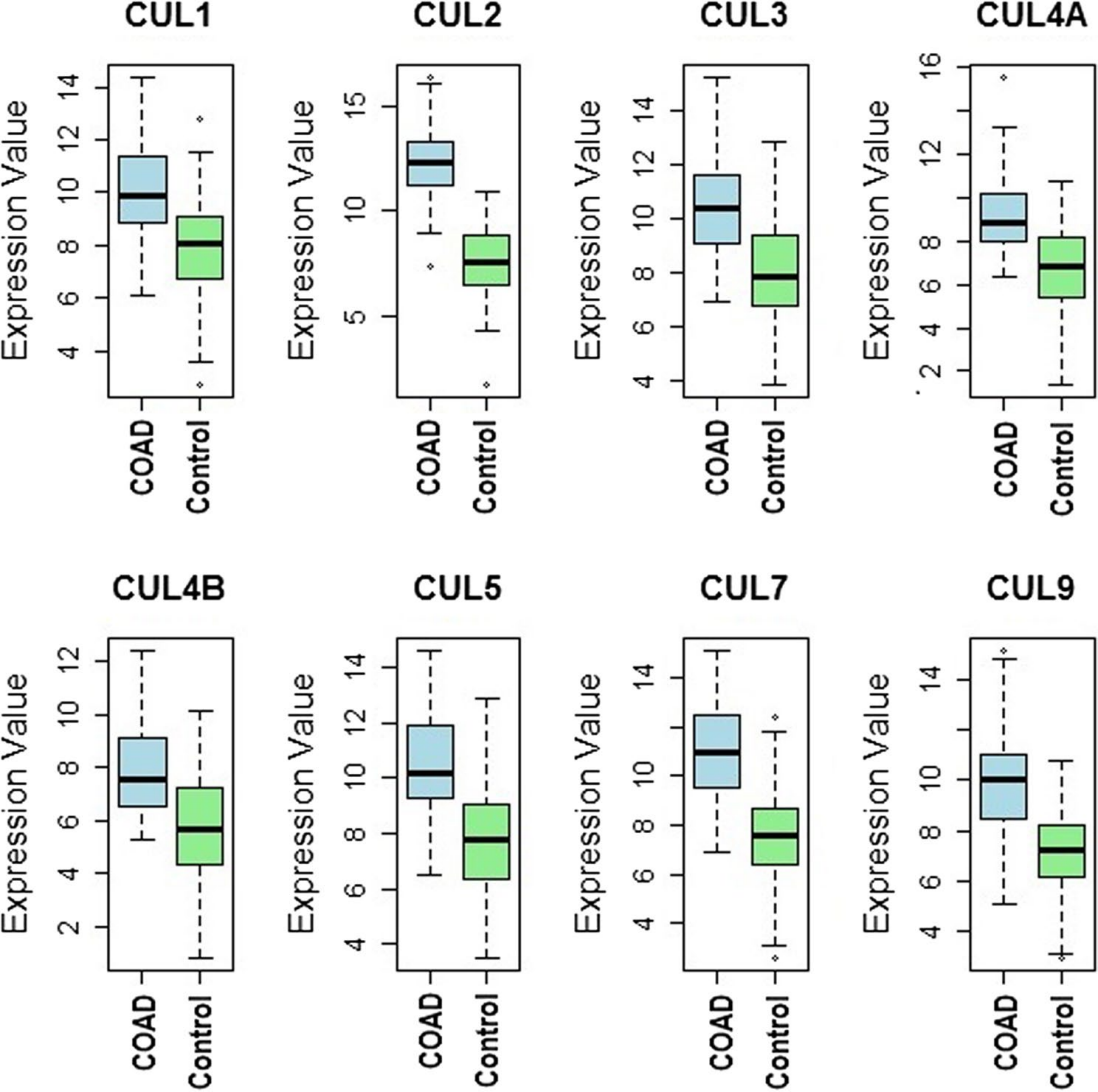
Validation of Cullin genes expression in colon cancer cell lines and normal control cell lines via the RT-qPCR assay. Box plots showing RT qPCR-based expression levels of CUL1, CUL2, CUL3, CUL4A, CUL4B, CUL5, CUL7, and CUL9 in COAD cell lines (*n* = 8) versus normal colon epithelial cell lines (*n* = 7). Each data point represents the mean of technical replicates for one biological replicate. Comparisons between cancer and normal groups were performed using an independent samples t test. Data are presented as mean ± SD from at least three independent biological experiments. P-value < 0.05

The expression levels of Cullin genes were further validated in COAD cell lines using RT-qPCR assays. As shown in Fig. 7, box plots compared the expression levels of CUL1, CUL2, CUL3, CUL4A, CUL4B, CUL5, CUL7, and CUL9 between the COAD cell line group (*n* = 8) and the control (normal colon epithelial) cell line group (*n* = 7). The analysis revealed a notable upregulation of all tested Cullin genes in the COAD cell lines compared to controls. Specifically, CUL1, CUL2, CUL3, CUL4A, CUL4B, CUL5, CUL7, and CUL9 exhibited significantly higher expression levels in the cancer cell lines, corroborating the transcriptomic findings obtained from database analyses (Fig. 7).

### Loss of CUL5 and CUL7 reduces proliferation, colony formation, and migration in SW480 and HCT116 cells

**Fig. 8:**
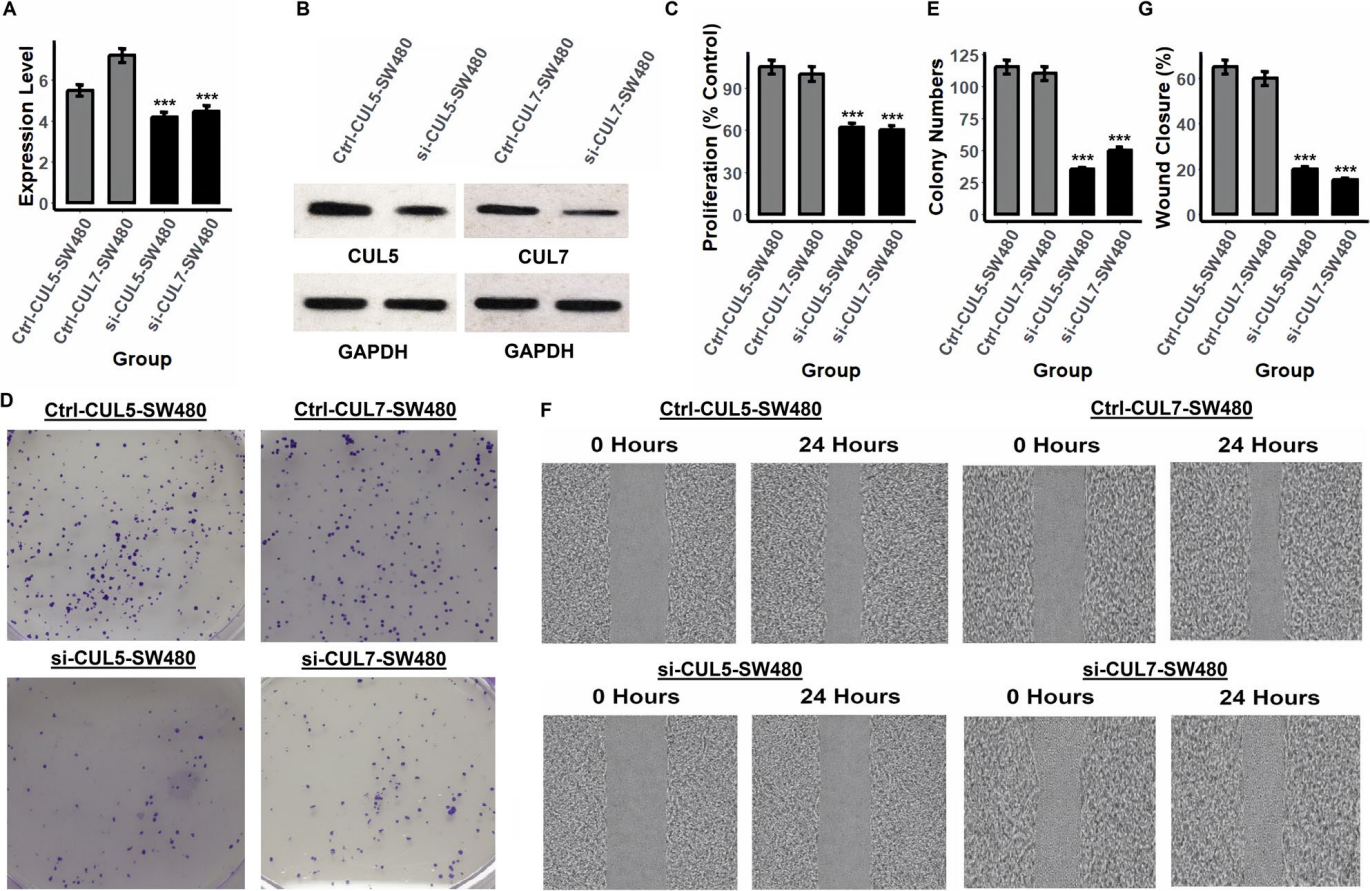
Knockdown of CUL5 and CUL7 suppresses proliferation, colony formation, and migration in SW480 colorectal cancer (CRC) cells. **A** RT-qPCR confirmed significant knockdown efficiency of CUL5 and CUL7 at the mRNA level. **B** Western blot analysis validated reduced protein expression of CUL5 and CUL7 compared to control siRNA (Ctrl). **C** Cell proliferation was significantly reduced in both si-CUL5 and si-CUL7-transfected cells. **D** Representative images from colony formation assays show decreased colony density following CUL5 or CUL7 silencing. **E** Quantification of colony numbers revealed a marked reduction upon knockdown. **F** Wound healing assay images at 0 and 24 h demonstrate impaired migration in si-CUL5- and si-CUL7-treated cells. **G** Wound closure was significantly reduced after knockdown compared to controls. Statistical comparisons were performed using an independent samples t test. For the time course proliferation data in panel **C**, two-way ANOVA followed by Tukey post hoc test was used. Data represent mean ± SD from at least three independent experiments. ***p-value < 0.001

**Fig. 9:**
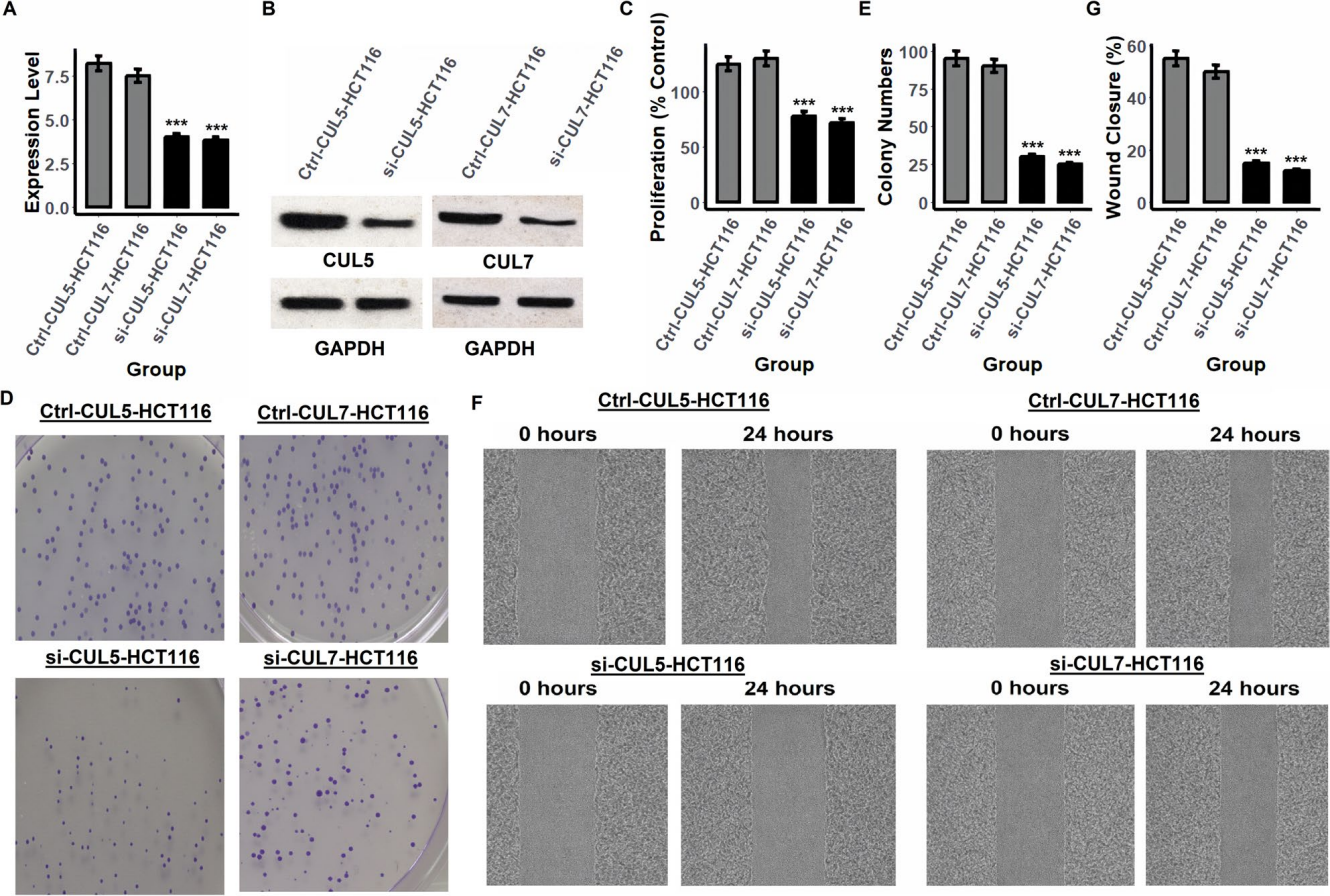
Silencing of CUL5 and CUL7 impairs tumorigenic properties of HCT116 colorectal cancer (CRC) cells. **A** RT-qPCR analysis confirmed efficient knockdown of CUL5 and CUL7 mRNA expression levels. **B** Western blot showed effective silencing of both proteins. **C** Cell proliferation was significantly reduced in both knockdown groups compared to controls. **D** Representative colony formation assay images illustrate diminished colony numbers upon CUL5 or CUL7 knockdown. **E** Quantitative analysis showed a significant decrease in colony numbers in si-CUL5- and si-CUL7-transfected cells. **F** Wound healing assays revealed reduced migratory ability following gene silencing at 24 h. **G** Quantification confirmed significant impairment of wound closure in knockdown groups relative to controls. Statistical comparisons were performed using an independent samples t test. For the time course proliferation data in panel C, two-way ANOVA followed by Tukey post hoc test was used. All data are shown as mean ± SD from at least three independent experiments. ***p-value < 0.001

CUL5 and CUL7 have emerged as potential prognostic markers in CRC based on their elevated expression and prognostic significance. To functionally validate their role in CRC progression, we performed siRNA-mediated knockdown of CUL5 and CUL7 in two representative CRC cell lines, including SW480 and HCT116 followed by a series of phenotypic assays assessing their impact on proliferation, colony formation, and cell migration (Figs. 8 and 9). Following siRNA transfection, qRT-PCR and Western blot analysis confirmed efficient knockdown of CUL5 and CUL7 at both transcript and protein levels in SW480 (Fig. 8A–B and supplementary data Fig. 1) and HCT116 (Fig. 9A–B and supplementary data Fig. 1) cells. These validations are critical to ensure that downstream phenotypic changes are attributable to target-specific gene silencing. In the cell proliferation assays, knockdown of either CUL5 or CUL7 led to a significant reduction in proliferation rates compared to control siRNA-transfected cells in both SW480 and HCT116 lines (Figs. 8C and 9C). These results indicate that CUL5 and CUL7 are required for optimal CRC cell growth and support their functional relevance in promoting tumor cell proliferation. Consistent with the proliferation data, colony formation assays revealed that CUL5 or CUL7 knockdown substantially impaired the clonogenic potential of CRC cells (Figs. 8D and 9D). Quantification showed a marked reduction in the number of colonies formed in si-CUL5 and si-CUL7 groups relative to controls (Figs. 8E and 9E), suggesting that these Cullin proteins contribute to long-term cell survival and replicative capacity. Further, wound healing assays demonstrated a significant defect in cell migratory ability following CUL5 or CUL7 knockdown. Compared to control groups, both SW480 and HCT116 cells exhibited delayed wound closure at 24 h post-scratch in the si-CUL5 and si-CUL7 conditions (Figs. 8F–G and 9F–G), indicative of compromised migratory function.

### ChIP-qPCR

**Fig. 10:**
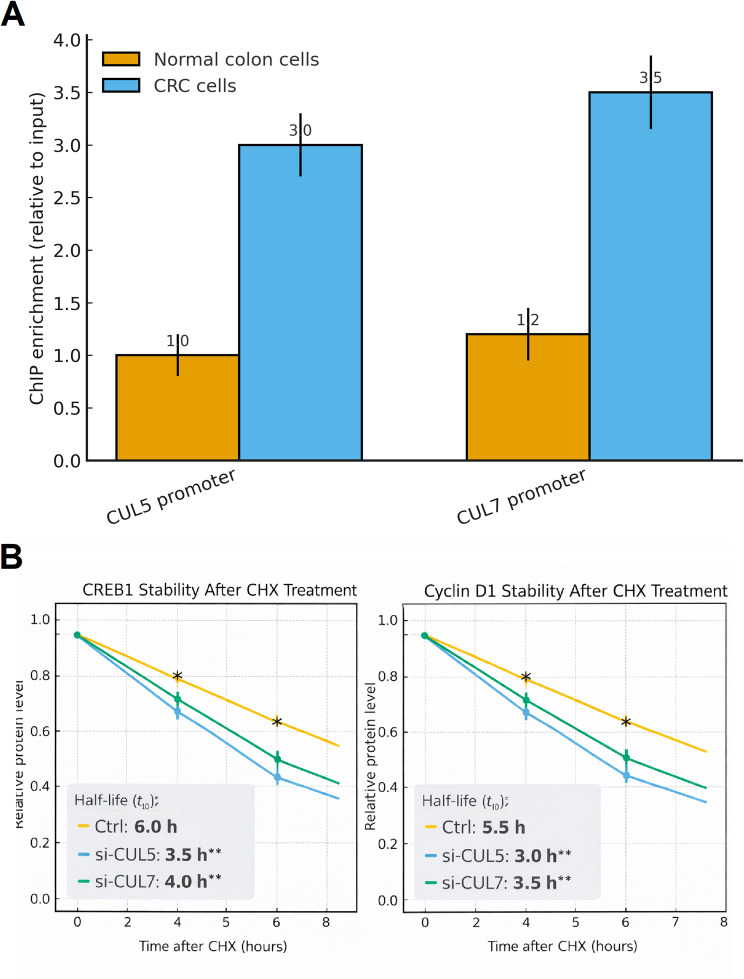
Epigenetic activation and post-translational regulation of CUL5 and CUL7 in colorectal cancer (CRC). **A** Increased H3K27ac enrichment at CUL5 and CUL7 promoters in CRC cells. ChIP-qPCR analysis showing elevated enrichment of the active histone mark H3K27ac at the promoter regions of CUL5 and CUL7 in CRC cells compared with normal colon epithelial cells. **B** CUL5 and CUL7 knockdown accelerates degradation of CREB1 and Cyclin D1 in CRC cells. Cycloheximide (CHX) chase assays were performed in SW480 and HCT116 cells. Data represent mean ± SD from three independent biological replicates, each performed in technical triplicate. Statistical comparisons between normal and CRC groups were performed using an independent-samples t test. *p-value < 0.05, **p-value < 0.01, and ***p-value < 0.001

To further investigate the transcriptional activation of CUL5 and CUL7 in COAD, we performed ChIP-qPCR using an antibody against the active promoter mark H3K27ac. The analysis revealed a pronounced increase in H3K27ac enrichment at both the CUL5 and CUL7 promoter regions in CRC cells (SW480 and HCT116) compared with normal colon epithelial cells (Fig. 10A). Specifically, CRC cells showed approximately three-fold higher enrichment at the CUL5 promoter and nearly four-fold enrichment at the CUL7 promoter relative to controls, indicating a substantially more open and transcriptionally active chromatin state at these loci (Fig. 10A). These findings support the notion that epigenetic activation of CUL5 and CUL7 promoters contributes to their up-regulated expression in CRC, consistent with the elevated mRNA levels observed in TCGA datasets and COAD cell lines.

### CUL5 and CUL7 knockdown accelerate CREB1 and Cyclin D1 degradation

To quantitatively evaluate whether CUL5 and CUL7 influence protein turnover dynamics, cycloheximide (CHX) chase assays were analyzed using first-order exponential decay modeling. As shown in Fig. 10B, log-transformed relative protein abundance declined approximately linearly over time across all experimental groups (R² > 0.95), supporting first-order degradation kinetics. In control (Ctrl) SW480 and HCT116 cells, CREB1 protein levels decreased to approximately 82%, 65%, 50%, and 40% of baseline at 2, 4, 6, and 8 h following CHX treatment, corresponding to an estimated half-life of approximately ~ 6.0 h. In contrast, silencing CUL5 reduced the estimated CREB1 half-life to approximately ~ 3.5 h, while CUL7 knockdown reduced it to approximately ~ 4.0 h (*p* < 0.01 vs. Ctrl; Fig. 10B), indicating accelerated CREB1 turnover upon depletion of either Cullin protein. Similarly, Cyclin D1 exhibited first-order decay kinetics under all conditions. In Ctrl cells, Cyclin D1 levels declined to approximately 80%, 62%, 48%, and 38% at 2, 4, 6, and 8 h, yielding an estimated half-life of approximately ~ 5.5 h. Knockdown of CUL5 reduced the Cyclin D1 half-life to approximately ~ 3.0 h, whereas CUL7 depletion shortened it to approximately ~ 3.5 h (*p* < 0.01 vs. Ctrl; Fig. 10B). These results demonstrate that loss of CUL5 or CUL7 accelerates degradation of CREB1 and Cyclin D1 and shortens their protein half-lives in CRC cells. Collectively, these kinetic analyses indicate that CUL5 and CUL7 are required to maintain normal turnover dynamics and steady-state protein levels of CREB1 and Cyclin D1 in colorectal cancer cells. While the precise ubiquitin-dependent mechanisms underlying these effects remain to be determined, the data clearly support a functional role for CUL5 and CUL7 in regulating the stability landscape of key proliferative and transcriptional regulators.

**Fig. 11:**
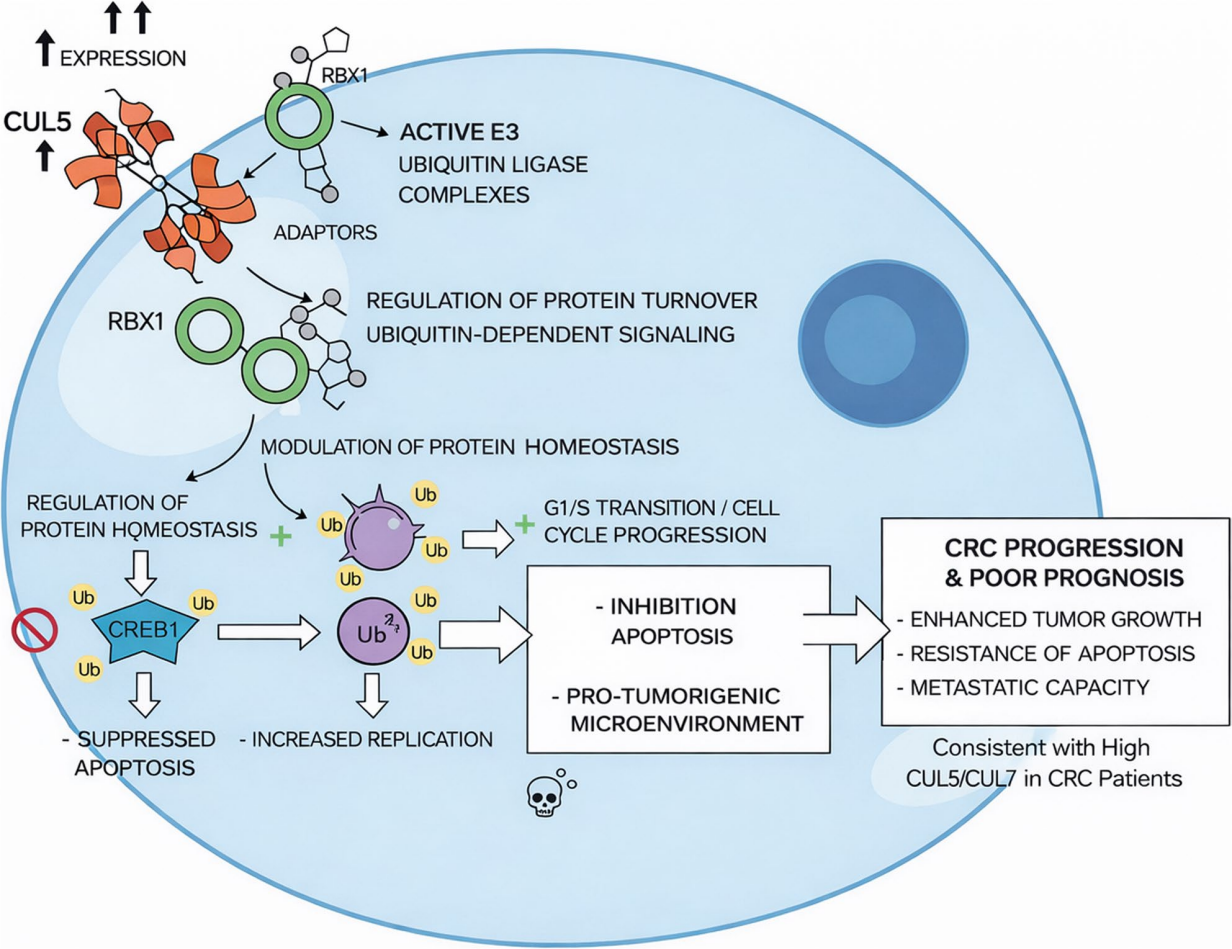
Proposed mechanistic model of CUL5 and CUL7-mediated colorectal cancer (CRC) progression. Schematic representation of the pathophysiological mechanism by which upregulated CUL5 and CUL7 contribute to CRC development and progression

To integrate our molecular and functional findings, we propose a refined regulatory model describing how elevated CUL5 and CUL7 expression contributes to CRC progression through modulation of ubiquitin-associated protein homeostasis (Fig. 11). CUL5 and CUL7 function as scaffold components of Cullin–RING E3 ubiquitin ligase (CRL) complexes, and their increased expression in CRC cells likely enhances the assembly and activity of specific CRL complexes in association with RBX1 and adaptor proteins. Our cycloheximide (CHX) chase analyses demonstrated that depletion of CUL5 or CUL7 significantly accelerated degradation of CREB1 and Cyclin D1, leading to reduced protein half-lives in CRC cells. These findings indicate that CUL5 and CUL7 are required to maintain steady-state protein levels of these key regulatory factors. Given that CRL complexes are classically associated with ubiquitin-mediated proteasomal degradation, the observed stabilization phenotype is unlikely to reflect a simple direct substrate-stabilizing mechanism. Instead, CUL5 and CUL7 may influence CREB1 and Cyclin D1 turnover indirectly through modulation of ubiquitination dynamics, regulation of upstream E3 ligases or deubiquitinases, alteration of adaptor specificity within CRL complexes, or broader effects on ubiquitin-dependent signaling networks. Consequently, our data support a model in which CUL5 and CUL7 contribute to the maintenance of CREB1 and Cyclin D1 protein homeostasis rather than directly mediating their non-degradative ubiquitination. Functionally, altered regulation of CREB1 may impact apoptosis-associated transcriptional programs, while modulation of Cyclin D1 stability promotes sustained G1/S cell-cycle progression, collectively enhancing proliferative capacity and tumor progression in CRC. Although ubiquitin-dependent signaling appears central to this regulatory axis, further studies are required to delineate the precise molecular intermediates and ubiquitin linkage types involved. Overall, these findings suggest that aberrant upregulation of CUL5 and CUL7 reshapes protein turnover networks in CRC cells, thereby sustaining oncogenic signaling and contributing to poor clinical outcomes.

## Discussion

CRC is one of the most prevalent malignancies worldwide, characterized by complex genetic and molecular alterations that drive its development and progression [31, 32]. The Cullin family of proteins, known for their role in ubiquitin-proteasome-mediated protein degradation, has been increasingly recognized for their involvement in cancer [33–35]. Our study aimed to elucidate the expression, prognostic significance, and functional impact of Cullin family genes in CRC.

The findings of our study revealed that the expression levels of CUL1, CUL2, CUL4A, CUL4B, CUL5, CUL7, and CUL9 are significantly elevated in CRC tissues compared to normal tissues. When Cullin genes are overexpressed, their increased activity can lead to the excessive degradation of tumor suppressor proteins, such as p27, p21, and RB1, thus promoting uncontrolled cell proliferation [36–38]. For instance, CUL1 is involved in the degradation of the cell cycle inhibitor p27 [39, 40], while CUL4A and CUL4B target the tumor suppressor p53 for degradation [41, 42]. Furthermore, the dysregulation of Cullin genes can disrupt DNA repair processes by degrading key regulatory proteins involved in DNA damage response, leading to genomic instability [43, 44]. The aberrant expression of these genes can also interfere with apoptosis, enabling cancer cells to evade programmed cell death [44]. Additionally, Cullin proteins are implicated in modulating various signaling pathways, such as the Wnt/β-catenin and NF-κB pathways, which are often dysregulated in cancers [45, 46]. Our expression analysis findings also align with previous studies demonstrating the up-regulation of these genes in various cancers. For instance, CUL1 has been shown to be overexpressed in breast cancer, promoting tumorigenesis through the regulation of cell cycle and apoptosis-related proteins [11, 47]. Similarly, elevated levels of CUL4A and CUL4B have been implicated in the progression of hepatocellular carcinoma and lung cancer, respectively [48–50]. Our findings further substantiate the role of these Cullin genes in CRC pathogenesis.

The findings of survival analysis revealed that high expression levels of CUL1, CUL2, CUL3, CUL4A, CUL4B, CUL5, CUL7, and CUL9 are associated with poorer survival outcomes in CRC patients, with significant prognostic values for CUL5 and CUL7. Previous studies have identified CUL5 and CUL7 as prognostic markers in other cancers, such as gastric and lung cancer [15, 30], where their elevated expression correlates with decreased overall survival. These findings suggest that CUL5 and CUL7 may serve as valuable prognostic biomarkers in CRC as well. Notably, our findings in CRC align with these observations, suggesting a conserved oncogenic role for both CUL5 and CUL7 across multiple cancer types. However, emerging evidence also highlights cancer-specific variability in their functions. For example, CUL5 has been reported to exert tumor-suppressive effects in certain breast cancer subtypes by promoting degradation of HER2, which contrasts with its oncogenic activity in CRC [51, 52]. Likewise, although CUL7 promotes proliferation and metastasis in CRC and neuroblastoma, some studies suggest that CUL7 loss enhances tumorigenesis in hepatocellular carcinoma, indicating context-dependent functions [15, 53]. These discrepancies emphasize the complexity of Cullin-mediated regulation and suggest that their biological impact may depend on cellular context, substrate availability, or differential activation of adaptor proteins. Collectively, the literature supports a predominantly oncogenic role for CUL5 and CUL7 in epithelial cancers, yet our findings highlight the need to interpret their functions within CRC-specific molecular landscapes.

Single-cell transcriptome analysis revealed that Cullin genes are associated with various cell types in the TME, including immune cells and fibroblasts. Dysregulation of Cullin genes can alter immune cell ratios, contributing to an immunosuppressive TME and promoting tumor progression. Studies have shown that Cullin genes, such as CUL4B, play a role in modulating the immune landscape in cancers by regulating the degradation of immune checkpoint proteins [19, 54].

Our findings demonstrate that loss of CUL5 and CUL7 significantly impairs proliferation, colony formation, and migration in CRC cells, supporting their functional role as oncogenic regulators. The observed phenotypic changes following siRNA-mediated knockdown in both SW480 and HCT116 cell lines highlight the importance of these Cullin family members in sustaining tumorigenic behaviors. These results are consistent with prior studies implicating CUL5 in promoting tumor survival by mediating the degradation of pro-apoptotic proteins such as NOXA, thereby facilitating evasion of apoptosis in CRC cells [55–57]. Similarly, CUL7 has been shown to regulate key oncogenic pathways and enhance cellular transformation and metastasis in multiple cancers, including CRC, where its high expression correlates with poor overall survival [58, 59]. The significant reduction in colony-forming ability and migration following knockdown suggests that CUL5 and CUL7 contribute not only to proliferation but also to long-term clonogenic potential and metastatic capacity—two critical hallmarks of cancer progression. Importantly, our CHX chase assays revealed that both CUL5 and CUL7 stabilize CREB1 and Cyclin D1—key regulators of proliferation and survival—providing a mechanistic explanation for their oncogenic effects. This stabilizing function has also been documented in other cancers [60–62], further supporting the conserved role of Cullin-RING ligases in maintaining oncogenic signaling.

Our study presents several advantages. It provides a comprehensive analysis of the expression and functional impact of Cullin family genes in CRC using extensive datasets from different online databases. The multi-faceted approach, including mRNA and protein expression analysis, survival analysis, mutational profiling, immune cell infiltration correlation, and drug sensitivity, offers a holistic view of the roles of these genes in CRC. The validation of findings through in vitro experiments further strengthens the conclusions. However, a few limitations should be acknowledged. The reliance on publicly available datasets may introduce biases related to data collection procedures, patient demographics, and institutional variability. In addition, bioinformatic analyses are inherently influenced by potential confounding factors, including batch effects arising from differences in sequencing platforms, sample processing protocols, or data integration pipelines. Although normalization algorithms were applied to minimize such variation, residual batch-related noise may still influence gene expression patterns or survival associations. Moreover, sample heterogeneity within bulk RNA-seq datasets represents another important limitation, as tumor samples comprise mixtures of malignant, stromal, and immune cell populations. This cellular complexity may obscure cell-type–specific expression differences. While the incorporation of single-cell RNA sequencing data partially addresses this issue, the limited number of CRC samples currently available in public scRNA-seq repositories may not fully capture inter-patient variability or tumor evolutionary diversity. From a mechanistic perspective, although CHX chase assays demonstrated that CUL5 and CUL7 regulate the stability of CREB1 and Cyclin D1, direct biochemical evidence of substrate ubiquitination was not performed in the present study. Specifically, endogenous ubiquitination assays or co-immunoprecipitation (Co-IP) experiments would be required to confirm physical interactions between CUL5/CUL7 and their proposed substrates and to establish whether ubiquitination occurs directly and through which linkage types. Therefore, while our findings strongly support a role for CUL5 and CUL7 in modulating protein stability, the precise ubiquitination mechanisms remain to be fully elucidated. Finally, additional functional rescue experiments, in vivo validation studies, and larger, multi-center patient cohorts will be necessary to confirm causality and to define the molecular context in which Cullin family members exert their oncogenic effects. Such studies will also be essential to evaluate the therapeutic feasibility of targeting Cullin-mediated pathways in CRC.

## Conclusion

his study comprehensively examined the expression patterns, clinical relevance, and functional significance of Cullin family genes (CUL1, CUL2, CUL3, CUL4A, CUL4B, CUL5, CUL7, and CUL9) in CRC using integrated bioinformatic analyses and in vitro experiments. We found that most Cullin genes were significantly up-regulated in CRC tissues, with higher expression associated with advanced tumor stage. Although survival trends were observed across several Cullin members, CUL5 and CUL7 emerged as the only genes with statistically significant prognostic value, highlighting their stronger association with CRC outcomes. Immunohistochemistry and mutational profiling further supported the involvement of Cullin genes in CRC biology, while single-cell analyses revealed cell type–specific expression patterns within the tumor microenvironment. Functional enrichment indicated their roles in ubiquitin-mediated proteolysis and cancer-associated signaling pathways. Importantly, experimental knockdown of CUL5 and CUL7 led to pronounced reductions in cell proliferation, colony formation, and migration, confirming their functional contribution to CRC progression. Mechanistic assays further demonstrated that loss of CUL5 or CUL7 accelerates the degradation of CREB1 and Cyclin D1, directly linking these Cullin proteins to the stabilization of key oncogenic regulators. These findings provide strong evidence that CUL5 and CUL7 promote CRC cell growth through post-translational control mechanisms. Collectively, our results suggest that CUL5 and CUL7 act as important drivers of CRC progression and hold potential as prognostic biomarkers and therapeutic targets. While additional in vivo studies are warranted, the current data establish a clear mechanistic and functional basis for their role in CRC.

## Supplementary Information

Supplementary Material 1.

## Data Availability

Any type of the data will be provided by the corresponding author.
