# Supplementary material for: A comprehensive analysis of the Cullin family reveals that CUL5 and CUL7 promote colorectal cancer progression and serve as prognostic markers
Source: Hereditas. 2026 Apr 22;163:71. doi: 10.1186/s41065-026-00677-8 (PMC13238039; doi:10.1186/s41065-026-00677-8)
Supplement: Supplementary file 1 — Supplementary Material 1. [file 41065_2026_677_MOESM1_ESM.pdf]

Supplementary data Table 1. Primer sequences used for amplification of GAPDH and Cullin gene family members.

Gene	Primer Type	Sequence (5′ → 3′)
GAPDH	Forward	TGACTTCAACAGCGACACCCA
GAPDH	Reverse	CACCCTGTTGCTGTAGCCAAA
CUL1	Forward	CAATGACGCTGGCTTTGTGGCT
CUL1	Reverse	CAAGGAGTCACAGTATCGAGCC
CUL2	Forward	GTCTTACTCCGTGCTGTGTCCA
CUL2	Reverse	CTGACTCCACAAATAGTGTTGGC
CUL3	Forward	TCGACAGCTCACACTCCAGCAT
CUL3	Reverse	GTGCTTCCGTGTATTAGAGCCAG
CUL4A	Forward	GTGCTTCCGTGTATTAGAGCCAG
CUL4A	Reverse	CTGTGGCTTCTTTGTTGCCTGC
CUL4B	Forward	GAAGCTACAGATGAAGAACTTGAG
CUL4B	Reverse	GCACTCTTTCCGACTAACAGGC
CUL5	Forward	CCTGATGCTGAACTTAGGAGGAC
CUL5	Reverse	GGTTCACTGAGAAGAGGGTACC
CUL7	Forward	CCGCAAACTCATCACCAACATCC
CUL7	Reverse	GGCACAGGTATCTGAGGAACAC
CUL9	Forward	GTGAGGACTCAAGCTACATGCC
CUL9	Reverse	CAGGTTCTCCAAGAGGATCACC

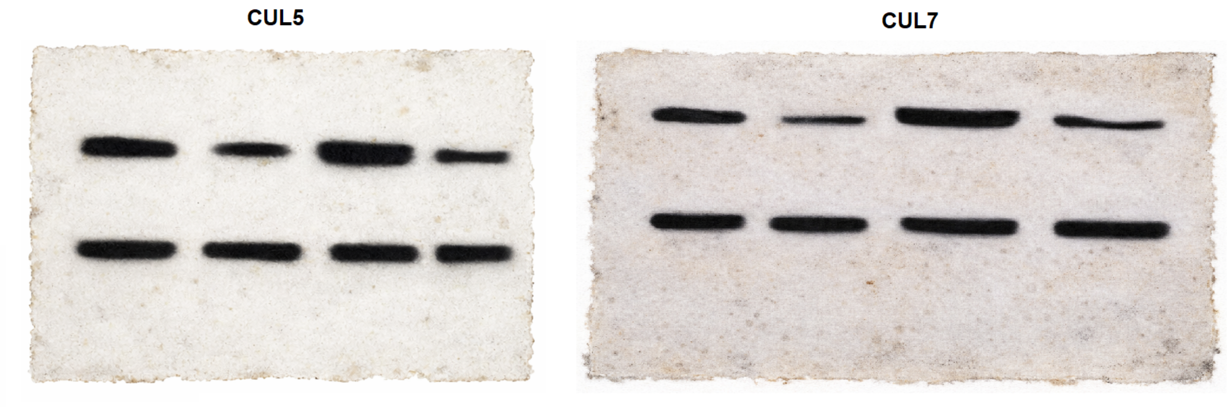

Supplementary data Figure 1: Uncut Western blot bands of CUL5, CUL7, and GAPDH.
